# The Performance, Energy and Nutrient Utilization, and Bone Mineralization of Broiler Chickens Fed Corn-Soybean Meal-Based Diets with Reduced Metabolizable Energy, Calcium, and Available Phosphorus Supplemented with Exogenous Enzymes

**DOI:** 10.3390/ani15223254

**Published:** 2025-11-10

**Authors:** Megan M. Bauer, Tuoying Ao, Jacqueline P. Jacob, Michael J. Ford, Anthony J. Pescatore, Ronan F. Power, Sunday A. Adedokun

**Affiliations:** 1 Department of Animal and Food Sciences, University of Kentucky, Lexington, KY 40546, USA; mmba242@yahoo.com (M.M.B.); jacquie.jacob@uky.edu (J.P.J.); michael.ford@uky.edu (M.J.F.); anthony.pescatore@uky.edu (A.J.P.); 2 Alltech Inc., Catnip Pike, Nicholasville, KY 40356, USA; tao@alltech.com (T.A.); rpower@alltech.com (R.F.P.)

**Keywords:** broiler chicken, corn-soybean meal, digestibility, exogenous enzyme, nutrient

## Abstract

This study evaluated the performance and energy and nutrient utilization of broiler chickens fed corn-soybean meal-based diets supplemented with a naturally fermented enzyme complex containing xylanase and phytase. This was evaluated using 300 one-day-old male Cobb broiler chicks in a randomized complete block design with five dietary treatments replicated 10 times with 6 chicks per replicate. The treatments consisted of a positive control (PC) group containing commercially recommended energy and nutrient levels, a negative control (NC) group with reduced metabolizable energy, calcium, and available phosphorus, and three levels of exogenous enzyme supplementation to the NC diet at 150, 200, 250 mg/kg, respectively. At the end of the trial, ileal digesta and excreta were collected for nutrient and energy digestibility and utilization determination, while tibia bones were collected for bone ash determination. The data was analyzed using Proc GLM of SAS 9.4 v 4. Enzyme supplementation quadratically increased (*p* < 0.05) average daily gain, average daily feed intake (ADFI), and feed efficiency during days 9–21. There was also a linear increase (*p* < 0.01) between enzyme level and ADFI during days 0–21. Compared with birds fed the PC diet, chickens fed the NC diet had lower (*p* < 0.01) utilization of DM, N, Ca, P, and energy, as well as lower (*p* < 0.01) apparent ileal digestibility of essential and non-essential amino acids (AA). Enzyme supplementation level showed a quadratic relation (*p* < 0.01) with the utilization of DM, N, Ca, P, and AMEn, as well as with the apparent ileal digestibility of essential and non-essential AA. A quadratic relationship was also observed for apparent ileal digestibility of DM, N, P, and digestible energy, except for Ca, where the relationship was linear (*p* < 0.001). Bone breaking strength and bone ash quadratically correlated (*p* < 0.05) with the level of enzyme supplementation. The results from this study indicated that the supplementation of exogenous enzyme to a corn–soybean meal-based diet resulted in benefits to performance, nutrient digestibility and utilization, and bone mineralization of broiler chicks compared to birds on the NC diet.

## Simple Summary

Feed is an important component of poultry production. In addition to constituting a significant portion of production cost, undigested and unabsorbed nutrients could undermine the health of the gastrointestinal tract as well as contribute to environmental pollution. Exogenous enzymes have been shown to improve energy and nutrient digestion in poultry. This study examined the role of an enzyme combo (phytase and xylanase) in improving energy and nutrient digestibility and utilization in 21-day-old broiler chickens fed corn–soybean meal-based diets. Enzyme supplementation improved average daily gain, average daily feed intake (ADFI), and feed efficiency, while a linear relationship was observed for ADFI. Enzyme supplementation showed a quadratic relationship with the utilization of DM, N, Ca, P, and AMEn, as well as with the apparent ileal digestibility of essential and non-essential amino acids.

## 1. Introduction

Corn and soybean meals are the most common feed ingredients used in commercial poultry diets. It is estimated that corn contributes about 65% of the energy in broiler diets [1], while 98% of the plant protein used in poultry feeds in the U.S. is from soybean meal (SBM) [2]. Although the nutrients contained in corn and SBM are generally considered to be highly digestible by animals, only about 50% of the energy in SBM can be utilized by poultry [3]. Also, 90% of the phosphorus (P) in corn and 75% of the P in SBM are present as phytate-P [4]. Due to the absence of adequate levels of endogenous phytase activities in poultry, only 30% of phytate-P can be utilized by poultry [5]. The undigested phytate in the gastrointestinal tract (GIT) has a strong antinutritive effect because it can bind cations such as Ca^2+^, Zn^2+^, Cu^2+^, and Mn^2+^ and form complexes with protein and starch to reduce availability of these nutrients [6,7,8].

Many exogenous enzymes are regularly supplemented to poultry diets to improve nutrient and energy digestibility and utilization. Among these enzymes, phytase and carbohydrase are the most widely used [9]. While phytase helps birds hydrolyze phytate, the carbohydrase digests the non-starch polysaccharides (NSP) in the plant-based feed ingredients, including corn and SBM [9,10,11]. It has been reported [12] that the supplementation of commercial enzyme preparation increased the starch utilization in ileal digesta and improved feed conversion of broilers fed corn-SBM-based diet. Silversides and Bedford [13] pointed out that enzyme treatment of corn-SBM-based diets can move starch digestion more proximally in the small intestine and increase the apparent metabolizable energy (AME) value of the diet.

There are conflicting reports on whether xylanase improves nutrient digestibility and reduces intestinal viscosity in primarily corn-based diets. Munyaka et al. [14] and Dunaway et al. [15] reported that although xylanase showed a greater digesta viscosity reduction in wheat diets, it was also able to reduce the viscosity of corn diets. However, Sing et al. [16] reported that xylanase supplementation to a corn-based diet did not significantly affect growth performance parameters. Rabello et al. [17] reported that while the addition of xylanase did not improve growth performance, it improved AME in broiler chickens’ fed corn-based diets. Similarly, Melo-Duran et al. [18] reported that broiler response to xylanase inclusion in corn-based diets varied based on the nutritional profile of the corn being fed. It has been reported that phytase, in addition to its positive effect on phosphorus digestibility, does exhibit extra-phosphoric effects, such as improvement in nitrogen (amino acids), energy, and dry matter (DM) digestibility [19,20,21].

Products with multi-enzyme activity have been created to enhance poultry’s ability to digest and utilize energy and nutrients in diets. The advantages of supplementing poultry diets with multi-enzyme combination include cost reduction, decreased risks of mixing errors by adding exogenous enzymes once rather than from multiple products, and, more importantly, the opportunity to exploit potential positive interactive effects among these enzymes. Allzyme^®^ Spectrum (Alltech Inc., Nicholasville, KY, USA) is an enzyme combo with both xylanase and phytase activities. This exogenous enzyme combo was supplemented to a negative control (NC) diet that contained reduced energy, calcium (Ca), and available P (avP), in anticipation of the enzymes increasing nutrient and energy release from their respective substrates. It is hypothesized that increasing supplementation of Allzyme^®^ Spectrum would lead to significant improvements in the response variables evaluated in this study. The objective of this study was to evaluate the effect of dietary supplementation of increasing levels of the enzyme combo on the growth performance, energy and nutrient digestibility and utilization, and bone (tibia) mineralization in 21-day-old broiler chickens.

## 2. Materials and Methods

### 2.1. Animal Housing, Management, and Experimental Design

Prior to the study, experimental procedures and management of birds were approved by the University of Kentucky’s Institutional Animal Care and Use Committee. A total of 300 one-day-old male broiler Cobb chicks were obtained from a commercial hatchery and fed a corn–SBM-based pre-starter diet (Table 1) between days 0 and 9 and were then moved to a corn–SBM-based starter diet (Table 2) for the remainder of the study (days 9–21). On day 0, the birds were weighed and randomly assigned to treatments in a randomized complete block design (RCBD) with cage location as the blocking factor. Each of the 5 dietary treatments consisted of 10 replicate cages of 6 chicks per cage. The experiment was conducted in battery cages (0.61 × 0.51 × 0.36 m) in an environmentally controlled room with 22 h of light and 2 h of darkness. Room temperature was monitored via two thermometers placed at two different locations in the room. Room temperature during the first week was 88 °F, which was reduced by 5 °F during the second (83 °F) and third (78 °F) weeks. All birds had free access to feed and water throughout the duration of the study.

### 2.2. Experimental Diets

Allzyme^®^ Spectrum, the enzyme used in this study, was supplied by Alltech Inc., (Nicholasville, KY, USA). The enzyme was analyzed for the activity levels of phytase and xylanase prior to diet formulation. The analyzed results showed that the enzyme contained 454,000 SPU/lb of phytase activity (*Aspergillus niger*) and 4.2 million XU/lb of xylanase activity (*Trichoderma longibrachiatum*). One solid-state fermentation phytase unit (SPU) is defined as the amount of enzyme that will release 1 mmol of inorganic P per minute under the conditions of the assay [22]. One xylanase unit is the amount of enzyme that will release 1 mmol of xylans per minute under the conditions of the assay. Enzyme activity was determined prior to the addition of the enzyme to the diets.

All the diets used in this study were corn–SBM-based. The positive control (PC) diets (pre-starter and starter phases) were the reference diets with energy and nutrients levels formulated to meet or exceed the recommended levels in the Cobb500™ Broiler performance and nutrition management guide, Siloam Springs, AR, USA [23] (Table 1 and Table 2). The negative control (NC) diets were formulated to contain reduced nutrient and energy (90 kcal less AME corrected for N [AMEn], 16.7% less Ca, and 33.3% less avP) relative to the PC diets (Table 1 and Table 2). The other three treatment diets were the NC diet supplemented with 150, 200, or 250 mg/kg of the enzyme. For each phase, the NC diet and the enzyme-supplemented diets were produced from a single basal diet and only supplemented with Allzyme^®^ Spectrum enzyme as needed. All starter diets contained titanium (Ti) dioxide (5 g/kg), which served as an indigestible marker for nutrient and energy digestibility and utilization determination.

### 2.3. Performance Assay and Sample Collection

All birds and feed were weighed per cage on days 0, 9, and 21 to determine performance response variables (average daily gain [ADG], average daily feed intake [ADFI], and feed efficiency [FE]). Daily mortality and body weight of dead bird(s) were recorded and used to adjust ADG and ADFI data using bird days.

Excreta samples were collected on days 20 and 21 and were dried at 55 °C in a forced-air oven for 5 days. The dried samples were ground using a Wiley Mill Laboratory Standard (Model No. 3, Arthur H. Thomas Co., Philadelphia, PA, USA) fitted with a 1 mm screen. Ground samples were stored in airtight plastic bags at 4 °C until analyzed for DM, gross energy (GE), (Ti), Ca, P, and nitrogen (N).

All birds within each cage were euthanized on day 21 and the right tibia from two birds with body weights closest to the cage average were removed and stored at −20 °C until processed for bone-breaking strength (BBS) and bone ash determination. Ileal digesta from the distal two-third of the ileum was collected from all the six birds within each cage by flushing with distilled water into clean pre-labeled plastic containers and stored at −20 °C, after which they were freeze-dried and ground using a coffee grinder prior to chemical analysis of DM, GE, Ti, amino acid (AA), Ca, P, and N concentrations.

### 2.4. Sample Analysis

Excreta and ileal digesta were analyzed in duplicates while diets were analyzed in triplicate. If the coefficient of variation fell above 5%, those analyses were repeated for that sample. The DM contents of the diets, ileal digesta, and excreta were determined by drying the sample at 105 °C for 24 h (method 934.01; [24]). The gross energy of the diets, ileal digesta, and excreta were determined using a bomb calorimeter (Parr adiabatic bomb calorimeter, model 6200, Parr Instruments, Moline, IL, USA) with the calibration standard being benzoic acid. The AA concentration of the diets and ileal digesta samples, the Ca, P, N, and Ti concentrations of the diets, ileal digesta, and excreta samples were determined at the Agricultural Experiment Station Chemical Laboratories, University of Missouri, Columbia, MO. The Ca concentration was analyzed using Inductively Coupled Plasma, and P concentration was analyzed using the gravimetric method [24]. The N concentration of the diets as well as the digesta and excreta samples were analyzed using the combustion method (model FP2000, LECO, St. Joseph, MI, USA) [24] with EDTA as the internal standard. Concentrations of AA were analyzed using method 982.30 E(a,b,c) [24], while the concentrations of Ti in the diet, excreta, and ileal digesta samples were determined as described by Myers et al. [25].

The tibias were cleaned thoroughly during collection and, following thawing, all remaining tissues and fat were removed. The BBS was measured using an Instron Materials tester (model 4301, Instron Corp., Canton, MA, USA) at a loading rate of 50 mm/min. Tibia samples were then dried for 24 h at 105 °C in an oven, (Precision Scientific Co., Chicago, IL, USA). After drying, the bones were then completely soaked in a glass jar with petroleum ether for four extraction periods each lasting for 24 h. Following the final extraction, after no color change in petroleum ether solution was observed, the bones were removed and allowed to dry for 24 h under the hood at room temperature. Details of the method used for tibia ash determination are provided in Bauer et al. [26].

### 2.5. Calculations and Statistical Analysis

Although all diets were individually analyzed for Ca, P, N, Ti, GE, and AA, the average of the analyzed values of the NC and all the 3 enzyme-supplemented diets were used for digestibility and utilization calculations because they were mixed from a single basal diet prior to the addition of the enzyme. The average of the analyzed values for the PC diet was used for digestibility and utilization calculations.

Apparent ileal nutrient and energy digestibility (AID) and energy and nutrient utilization (NU) of DM, energy, N, Ca, and P were calculated as described by Adejumo et al. [27]:

AID or NU (%) = [1 − (Ti_I_/Ti_o_) × (n_o_/n_I_)] × 100.

In this equation, Ti_I_ is the titanium concentration in the diet (%), Ti_o_ is the concentration of titanium in the ileal digesta or excreta (%), n_o_ is the concentration of energy or nutrient in ileal digesta or excreta (%), and n_I_ is the concentration of energy or nutrient in the diet (%).

The AME and apparent digestible energy (ADE) were calculated using the equation below:

AME or ADE, kcal/kg = Calculated energy utilization or ileal energy digestibility (%) × GE of the diet (kcal/kg). The AMEn was obtained by correcting AME for N using the equation provided by Hills and Anderson [28].

The data were analyzed with PROC GLM of SAS 9.4 v 4 [29], appropriate for a RCBD. PROC IML was used to generate the coefficients used for orthogonal polynomial contrasts. Prior to statistical analysis, data that deviated from first or third quartiles by more than 3 times the interquartile range within each dietary treatment were removed. Orthogonal polynomial contrasts were used to determine the effects of increasing dietary levels of exogenous enzyme supplementation, while simple contrasts were performed between the PC and NC diets and between the PC and enzyme-supplemented diets. The digestibility and utilization data were presented on a DM basis.

## 3. Results

### 3.1. Nutrient Content of Experimental Diets

The experimental diets used in this study were corn–SBM-based and consisted of the pre-starter (days 0–9) and the starter (days 9–21) diets. Both sets of diets were analyzed for N, GE, Ca, P, Lys, Met, Cys, and Thr. The formulated and analyzed values are presented in Table 1 (pre-starter diet, days 0–9) and Table 2 (starter diet, days 9–21). For the starter diets, the formulated values for CP (N × 6.25), Ca, and P in the PC diet were 19.5, 0.80, and 0.64%, respectively, while the corresponding values for the NC diet for CP, Ca, and P were 19.5, 0.65, and 0.51%, respectively. For both diets (pre-starter and starter), the formulated and analyzed values followed the expected trends.

### 3.2. Growth Performance

The growth performance data are presented in Table 3 and Table 4. Chickens fed the NC diet had lower (*p* < 0.05) ADFI compared to those fed the PC diet (days 9–21 and 0–21). Increasing level of enzyme supplementation resulted in a linear increases (*p* < 0.05) ADFI (days 9–21 and 0–21) and quadratic responses (*p* < 0.05) in ADG (days 9–21). The ADFI between the PC and enzyme-supplemented diets were different (days 9–21 and 0–21; *p* < 0.05) with birds on enzyme-supplemented diet having lower (*p* < 0.05) ADFI compared to birds on the PC diet. Exogenous enzyme supplementation quadratically influenced FE (*p* < 0.05; days 9–21).

### 3.3. Energy and Nutrient Digestibility and Utilization

Apparent ileal DM, N, P, and energy digestibility, as well as ADE, were lower (*p* < 0.05) in birds fed the NC diet compared with birds fed the PC diet, while increasing levels of exogenous enzyme supplementation to the NC diet resulted in quadratic responses (*p* < 0.05) for these response variables (Table 5). Comparison between the PC and enzyme-supplemented diets showed that birds on diets containing enzymes had higher (*p* < 0.05) Ca and P digestibility, while the increasing levels of enzyme supplementation to the NC diet resulted in a linear effect on Ca digestibility and a quadratic effect on P digestibility (*p* < 0.05; Table 5). Birds fed the NC diet had lower (*p* < 0.01) AMEn and utilization of DM, N, Ca, P, and energy compared with birds fed the PC diet (Table 6). Birds fed enzyme-supplemented diets had higher (*p* < 0.05) DM, N, Ca, and P utilization compared to those fed the PC diet. Increasing levels of enzyme supplementation to the NC diet resulted in a quadratic response (*p* < 0.01) for AMEn and DM, N, Ca, and P utilization (Table 6).

Apparent ileal essential and non-essential AA digestibility were lower in birds fed the NC diet (*p* < 0.01) compared with those fed the PC diet (Table 7 and Table 8). No difference was observed in apparent ileal AA digestibility between birds fed the PC and the enzyme supplemented diets. Quadratic relationships (*p* < 0.05) were observed for dietary enzyme supplemental level and AA digestibility (Table 7 and Table 8).

### 3.4. Bone Mineralization

The effects of increasing exogenous enzyme supplementation on tibia bone mineralization are reported in Table 9. Birds fed the NC diet had lower (*p* < 0.01) BBS and percent tibia ash compared with birds fed the PC diet. Enzyme supplementation had a quadratic relationship (*p* < 0.01) with both BBS and tibia bone ash.

## 4. Discussion

Although corn contains relatively less NSP compared to the levels found in other grains such as wheat and barley, the chemical composition and nutritional value of corn have been proved to vary [1] and could be improved by supplemental carbohydrase enzymes [30,31,32]. However, SBM contains a fair amount of highly soluble NSP [10,33] and could benefit from supplemental carbohydrase enzymes. The Majority of P in corn and SBM are part of the phytic acid structure, which negatively impacts the availability of P as well as many other nutrients, including AA and several minerals, to poultry. In the current study, although the ADFI of birds fed enzyme-supplemented diets was lower than that of birds fed the PC diet (days 9–21 and 0–21), a linear increase in ADFI was observed with increasing enzyme supplementation (days 9–21). In addition to this, quadratic increase in ADFI, ADG, and FE were observed (days 9–21). These improvements in performance as a result of enzyme supplementation are similar to what has been previously reported in the literature [26]. The quadratic effect, which may indicate a slowdown in the rate of improvement (days 9–21 ADFI and ADG) with increasing levels of supplementation—or even a decrease in improvement compared with lower inclusion levels (e.g., FE days 9–21)—could be attributed to a reduced availability of substrate for the enzyme to act upon. An enzyme, whether endogenous or exogenous, cannot function in isolation; it requires an appropriate substrate. When most of the substrates have been utilized, the efficiency with which the enzyme performs its function diminishes. This noticeable improvement in performance could be attributed to the birds’ enhanced ability to digest and utilize the nutrients and energy released by the phytase and xylanase in the enzyme complex supplemented to the NC diet. Furthermore, the improvement in performance could be attributed to the enhanced nutrient and energy digestibility and utilization resulting from exogenous enzyme supplementation, which produced quadratic increases in energy and all evaluated nutrients, except for Ca digestibility, where the effect was linear. For instance, enzyme supplementation to the NC diet improved P and Ca digestibility by 36.4 and 24.5%, respectively, while the corresponding increases in P and Ca utilization were 43.6 and 38.2%, respectively. The results from the current study agree with those reported by [1,26,34,35,36]. However, some studies have reported no beneficial effects of enzyme complexes—including xylanase and β-glucanase—on the performance of broilers fed corn–SBM-based diet [16,17,37]. The positive effects in the current study may attributed to the Allzyme^®^ Spectrum, which contains xylanase and phytase capable of breaking down the cell wall matrix, particularly the insoluble components, thereby facilitating the release of nutrients encapsulated within cell walls [38,39].

The higher levels of N utilization and AID with enzyme supplementation agree with many published reports. Gonzalez-Ortiz et al. [40] found that inclusion of xylanase and phytase improved N retention in broilers, while Gallardo et al. [41] reported that inclusion of phytase and carbohydrase enzymes improved ileal digestibility of AA in broiler chickens fed corn-SBM-based diet. Similarly, researchers [36,42] reported an improvement in N utilization and ileal AA digestibility following phytase supplementation of corn-SBM-based diets. In contrast, Woyengo et al. [43] found no beneficial effects of phytase supplementation on apparent ileal AA digestibility in broilers. Such inconsistencies in phytase effects on N and AID of AA could be due to differences in diet composition (e.g., ingredient composition affecting digesta viscosity), CP or AA deficiencies, bird age, or enzyme inclusion level. Any or all of these factors could influence the outcomes of exogenous enzyme supplementation in broiler diets. The responses of AME and ADE to enzyme supplementation in this study correspond with those reported by Peniazek et al. [44], who observed significant increases in the AME of an NC diets supplemented with enzymes. Similar findings were also reported [1,45].

In the present study, BBS and tibia ash decreased by 30.4% and 5.8%, respectively, in birds fed the NC diet compared with those fed the PC diet. Increasing levels of enzyme supplementation to the NC diet improved (average value) BBS and tibia ash by 37.5% and 6.7%, respectively. These improvements were expected, since phytase hydrolyzes phytate to release P and phytate-bound mineral complexes, thereby increasing mineral availability to the birds. This aligns with the observed improvements in P and Ca digestibility and utilization. Although the direct link between phytase supplementation and bone mineralization is well established, it is noteworthy that a tendency (*p* = 0.06) for xylanase to improve (9.4%) tibia breaking strength has also been reported [46]. Improved digestibility and utilization of nutrients likely contributed to enhanced bone mineralization. Furthermore, Bauer et al. [26] reported similar findings with the same enzyme complex in wheat–SBM-based diets. These results also agree with those of Leyva-Jimenez et al. [47], who showed phytase addition improved bone quality, including ash and breaking strength, in broilers. Similarly, Walters et al. [36] demonstrated that phytase supplementation to low-P diets improved tibia quality parameters compared to birds fed the NC diet.

Overall, the results from this study clearly indicate that dietary supplementation of the exogenous enzyme complex to corn-SBM-based diets resulted in improved performance, nutrient and energy digestibility and utilization and bone mineralization, despite the lower levels of AMEn, Ca, and avP levels in the NC diet. For example, comparing the average values of the NC + enzyme treatments with their respective PC treatments showed the following improvements: P digestibility (64.5 vs. 55.6%); P utilization (64.6 vs. 58.9%); Ca digestibility (61.0 vs. 46.1%); and Ca utilization (68.4 vs. 58.3%). These improvements in performance, nutrient, and energy digestibility and utilization occurred despite the reduced energy, Ca, and avP levels in the NC diet. The ability of exogenous enzymes to enhance energy and nutrient digestion and utilization provides significant economic and environmental benefits.

## 5. Conclusions

The results from this study indicate that supplementing a corn-SBM-based diet that was formulated to contain reduced energy and nutrients (Ca and aP) with an exogenous enzyme complex containing phytase and xylanase improved growth performance, energy, and nutrients digestibility and utilization in 21-d-old broiler chickens. Most of the response variables measured in broiler chickens fed the low-energy and nutrient-deficient diets supplemented with increasing levels of enzyme complex were not different from those of birds fed the PC diet. This feeding strategy may help producers lower feed costs and reduce nutrient excretion into the environment.

## Figures and Tables

**Table 1:** Ingredient and nutrient composition of the experimental pre-starter diets fed to broiler chickens from days 0–9 (%, on as-fed basis) ^1^.

Ingredient	Positive Control	Negative Control ^2^
	Treatment A	Treatment B
Corn	62.42	59.30
Soybean meal (47% CP)	33.32	32.21
Wheat bran	0.00	5.23
Soy oil	0.28	0.00
L-Lysine HCl	0.32	0.33
DL-Methionine	0.29	0.29
Salt (NaCl)	0.41	0.41
Limestone	1.06	1.17
Dicalcium phosphate	1.67	0.83
Vitamin–mineral premix ^3^	0.15	0.15
Choline chloride (60%)	0.08	0.08
Total	100.0	100.0
Formulated nutrient and energy content		
AMEn, kcal/kg	2975	2885
CP, %	21.5	21.5
Ca, %	0.90	0.75
Total P, %	0.71	0.59
Available P, %	0.45	0.30
SID AA ^4^		
Lys, %	1.20	1.20
Met, %	0.55	0.55
Met + Cys, %	0.80	0.80
Thr, %	0.66	0.67
Analyzed nutrient and energy content		
CP, %	20.5	20.8
Gross energy, kcal/kg	3947	3897
Ca, %	0.99	0.77
Total P, %	0.69	0.57
Lys, %	1.44	1.43
Met, %	0.58	0.55
Met + Cys, %	0.88	0.86
Thr, %	0.76	0.76

^1^ The three enzyme-containing dietary treatments (C, D, and E) were mixed from the same negative control basal diet. Exogenous enzyme (Allzyme^®^ Spectrum) was added to the basal diet at the rates of 150, 200, and 250 mg/kg to produce diets containing increasing levels of enzyme activity. Allzyme^®^ Spectrum contains 1 million SPU phytase activity (*Aspergillus niger*) and 9.26 million XU xylanase activity (*Trichoderma longibrachiatum*)/kg. One solid state fermentation phytase unit (SPU) is defined as the amount of enzyme that will release 1 mmol of inorganic P per minute under the conditions of the assay. One xylanase unit is the amount of enzyme that will release 1 mmol of xylans per minute under the conditions of the assay. The calculated enzyme activity for each enzyme containing diet is 150 SPU and 1389 XU, 200 SPU and 1852 XU, and 250 SPU and 2315 XU for the 150, 200, and 250 mg/kg rates, respectively. ^2^ The negative control diet and diets containing supplemental exogenous enzymes (C, D, and E) were mixed from a single basal diet. ^3^ The following quantities of vitamins and microminerals per kg of the complete diet were provided: vitamin A (retinyl acetate), 8820 IU; vitamin D_3_ (cholecalciferol), 2822 IU; vitamin E (_dl_-α-tocopheryl acetate), 26 IU; vitamin K, 0.73 mg; thiamine, 1.76 mg; riboflavin, 6.17 mg; pantothenic acid, 14 mg; niacin, 44 mg; pyridoxine, 4 mg; folic acid, 0.88 mg; biotin, 0.18 mg; vitamin B_12_, 0.02 mg; choline, 480 mg and 32 mg of iron from iron sulfate; 8 mg of copper from copper sulfate; 51 mg of manganese from manganese oxide; 60 mg zinc from zinc oxide; 1.48 mg iodine from ethylenediamine dihydroiodide; and 0.24 mg selenium from sodium selenite. ^4^ Standardized ileal digestibility.

**Table 2:** Ingredient and nutrient composition of the experimental starter diets fed to broiler chickens from day 9–21 (%, on as-fed basis) ^1^.

Ingredient	Positive Control	Negative Control
	Treatment A	Treatment B ^2^
Corn	66.55	65.60
Soybean meal (47% CP)	28.35	27.40
Wheat bran	0.00	3.49
Soy oil	0.90	0.00
L-Lysine HCl	0.37	0.38
DL-Methionine	0.29	0.29
L-Threonine	0.04	0.05
Salt (NaCl)	0.39	0.39
Limestone	0.98	1.10
Dicalcium phosphate	1.43	0.60
Vitamin–mineral premix ^3^	0.15	0.15
Choline chloride (60%)	0.08	0.08
Titanium dioxide	0.50	0.50
Total	100.0	100.0
Formulated nutrient and energy content		
AMEn, kcal/kg	3057	2967
CP, %	19.5	19.5
Ca, %	0.80	0.65
Total P, %	0.64	0.51
Available P, %	0.40	0.25
SID ^4^ amino acid, %		
Lys	1.13	1.13
Met	0.53	0.53
Met + Cys	0.76	0.76
Thr	0.60	0.61
Analyzed nutrient and energy content		
CP, %	19.2	18.8
Gross energy, kcal/kg	3943	3908
Ca, %	0.73	0.67
Total P, %	0.60	0.50
Lys, %	1.38	1.36
Met, %	0.49	0.52
Me + Cys, %	0.78	0.81
Thr, %	0.76	0.76

^1^ The three enzyme-containing dietary treatments (C, D, and E) were mixed from the same negative control basal diet. Exogenous enzyme (Allzyme^®^ Spectrum) was added to the basal diet at the rates of 150, 200, and 250 mg/kg to produce diets containing increasing levels of enzyme activity. Allzyme^®^ Spectrum contains 1 million SPU phytase activity (*Aspergillus niger*) and 9.26 million XU xylanase activity (*Trichoderma longibrachiatum*) per kg. One solid state fermentation phytase unit (SPU) is defined as the amount of enzyme that will release 1 mmol of inorganic P per minute under the conditions of the assay. One xylanase unit is the amount of enzyme that will release 1 mmol of xylans per minute under the conditions of the assay. The calculated enzyme activity for each enzyme containing diet is 150 SPU and 1389 XU, 200 SPU and 1852 XU, and 250 SPU and 2315 XU for the 150, 200, and 250 mg/kg rates, respectively. ^2^ The negative control diet and diets containing supplemental exogenous enzymes (C, D, and E) were mixed from a single basal diet. ^3^ The following quantities of vitamins and microminerals per kg of complete diet were provided: vitamin A (retinyl acetate), 8820 IU; vitamin D_3_ (cholecalciferol), 2822 IU; vitamin E (_dl_-α-tocopheryl acetate), 26 IU; vitamin K, 0.73 mg; thiamine, 1.76 mg; riboflavin, 6.17 mg; pantothenic acid, 14 mg; niacin, 44 mg; pyridoxine, 4 mg; folic acid, 0.88 mg; biotin, 0.18 mg; vitamin B_12_, 0.02 mg; choline, 480 mg and 32 mg of iron from iron sulfate; 8 mg of copper from copper sulfate; 51 mg of manganese from manganese oxide; 60 mg zinc from zinc oxide; 1.48 mg iodine from ethylenediamine dihydroiodide; 0.24 mg selenium from sodium selenite. ^4^ Standardized ileal digestibility.

**Table 3:** Effect of increasing dietary enzyme supplementation levels on the performance of 21-day-old broiler chickens fed corn–soybean meal-based diets ^1,2^.

Treatment	Diet	Allzyme Spectrum, mg/kg	IBW, g/b	Feed Intake, g/b/d			Body Weight Gain, g/b/d		
				d 0–9	d 9–21	d 0–21	d 0–9	d 9–21	d 0–21
A	PC	0	40.7	21.3_z_	74.2	52.6	17.6_z_	47.6	35.2
B	NC	0	40.4_z_	21.2	68.3	48.6	17.3_z_	43.1	32.1
C	NC	150	40.8	22.2	70.0	50.4	18.3	45.0_z_	33.5
D	NC	200	41.1_z_	20.7	70.1_z_	48.1	16.9	46.7_z_	32.2
E	NC	250	40.7	22.2_z_	73.3_z_	52.3_z_	17.5	47.1	34.8
Pooled standard deviation			0.63	1.32	2.43	2.77	1.71	3.11	3.38
*p*-value			0.187	0.069	0.002	0.001	0.446	0.015	0.147
			--------------------------------Probability----------------------------------						
PC vs. NC			0.309	0.844	<0.001	0.003	0.684	0.002	0.047
PC vs. Enzyme-supplemented diets			0.448	0.435	0.018	0.029	0.964	0.235	0.176
Linear effect of enzyme supplementation			0.204	0.863	0.004	0.002	0.737	0.230	0.061
Quadratic effect of enzyme supplementation			0.035	0.262	0.024	0.564	0.503	0.003	0.504

^1^ PC = Positive control; NC = Negative control; IBW = Initial body weight. Pre-starter diet fed between d 0 and 9 and starter diet fed between d 9 and 21. ^2^ n = 10 replicate cages with 6 birds/replicate, except for subscript z where n = 9.

**Table 4:** Effect of increasing dietary enzyme supplementation levels on feed efficiency in 21-day-old broiler chickens fed corn–soybean meal-based diets ^1,2^.

Treatment	Diet	Allzyme^®^ Spectrum, mg/kg	Feed Efficiency		
			Days 0–9	Days 9–21	Days 0–21
A	PC	0	0.831	0.641	0.668
B	NC	0	0.808_z_	0.628_z_	0.666_z_
C	NC	150	0.826	0.632	0.663
D	NC	200	0.813	0.661	0.681_z_
E	NC	250	0.824_z_	0.652	0.684_z_
Pooled standard deviation			0.04	0.02	0.03
*p*-value			0.628	0.020	0.339
			--------------------------------Probability----------------------------------		
PC vs. NC			0.182	0.228	0.827
PC vs. Enzyme-supplemented diets			0.471	0.402	0.433
Linear effect of enzyme supplementation			0.352	0.281	0.524
Quadratic effect of enzyme supplementation			0.617	0.004	0.225

^1^ PC = Positive control; NC = Negative control. Pre-starter diet fed between d 0 and 9 and starter diet fed between d 9 and 21. ^2^ n = 10 replicate cages with 6 birds/replicate, except for subscript z where n = 9.

**Table 5:** Effect of increasing enzyme supplementation on apparent ileal nutrient and energy digestibility in 21-day-old broiler chickens fed corn–soybean meal-based diets (DM basis) ^1,2^.

Treatment	Diet	Allzyme^®^ Spectrum, mg/kg	DM, %	N, %	Ca, %	P, %	Energy, %	ADE, Kcal/kg
A	PC	0	79.7	86.5	46.1_z_	55.6_z_	81.3_z_	3561_z_
B	NC	0	74.3	83.1	49.0	47.3	76.7	3328
C	NC	150	80.1	87.1	63.8_z_	61.5_z_	81.8	3550_z_
D_z_	NC	200	79.0	86.9	62.1	64.3	80.9	3510
E_z_	NC	250	80.1	87.5	57.2	67.8	81.7	3549
Pooled standard deviation			1.95	1.67	9.05	5.37	1.73	75.26
*p*-value			<0.001	<0.001	<0.001	<0.001	<0.001	<0.001
			--------------------------------Probability----------------------------------					
PC vs. NC			<0.001	<0.001	0.451	0.002	<0.001	<0.001
PC vs. Enzyme-supplemented diets			0.909	0.271	<0.001	<0.001	0.777	0.403
Linear effect of enzyme supplementation			0.716	0.462	<0.001	0.001	0.882	0.235
Quadratic effect of enzyme supplementation			<0.001	<0.001	0.210	<0.001	<0.001	<0.001

^1^ PC = Positive control; NC = Negative control; DM = dry matter; N = nitrogen; Ca = calcium; P = phosphorus; ADE = apparent ileal digestible energy. Pre-starter diet fed between d 0 and 9 and starter diet fed between d 9 and 21. ^2^ n = 10 replicate cages with 6 birds/replicate, except for subscript z where n = 9.

**Table 6:** Effect of increasing level of enzyme supplementation on apparent nutrient and energy utilization in 21-day-old broiler chickens fed corn–soybean meal-based diets (DM basis) ^1,2^.

Treatment	Diet	Allzyme^®^ Spectrum mg/kg	DM, %	N, %	Ca, %	P, %	Energy, %	AME, kcal/kg	AMEn, Kcal/kg
A	PC	0	76.4	61.9	58.3	58.9	78.7	3446	3351
B	NC	0	66.8	51.7	49.5	45.0	69.7	3024	2853
C	NC	150	77.1	65.0	66.8_z_	64.0	79.0	3429	3305
D	NC	200	77.3	64.7	69.0_z_	65.3_z_	79.1	3436	3312
E	NC	250	77.6_z_	65.3	69.5	64.6	79.5_z_	3451_z_	3336
Pooled standard deviation			1.17	4.13	4.63	3.24	1.06	46.15	54.31
*p*-value			<0.001	<0.001	<0.001	<0.001	<0.001	<0.001	<0.001
			--------------------------------Probability----------------------------------						
PC vs. NC			<0.001	<0.001	<0.001	<0.001	<0.001	<0.001	<0.001
PC vs. Enzyme-supplemented diets			0.031	0.045	<0.001	<0.001	0.172	0.657	0.101
Linear effect of enzyme supplementation			0.065	0.081	<0.001	<0.001	0.316	0.497	0.060
Quadratic effect of enzyme supplementation			<0.001	<0.001	<0.001	<0.001	<0.001	<0.001	<0.001

^1^ PC = Positive control; NC = Negative control; DM = dry matter; N = nitrogen; Ca = calcium; P = phosphorus; AME = apparent metabolizable energy; AMEn = apparent metabolizable energy corrected for nitrogen. Pre-starter diet fed between d 0 and 9 and starter diet fed between d 9 and 21. ^2^ n = 10 replicate cages with 6 birds/replicate, except for subscript z where n = 9.

**Table 7:** Effect of increasing level of enzyme supplementation on apparent ileal digestibility of essential amino acids in 21-day-old broiler chickens fed corn–soybean meal-based diets (DM basis) ^1,2^.

Treatment	Diet	Allzyme^®^ Spectrum, mg/kg	Arg, %	His, %	Ile, %	Leu, %	Lys, %	Met, %	Phe, %	Thr, %	Trp, %	Val, %
A	PC	0	91.6	90.0	87.1	88.8	91.4	94.5	88.6	83.7	89.7	86.3
B	NC	0	89.1	87.0	84.4	86.0	89.6	93.4	85.5	79.3	86.1	83.1
C	NC	150	92.0	90.4	88.4	89.7	92.1	95.0	89.6	85.0	89.6	87.3
D	NC	200	91.8	90.3	88.1	89.4	92.0	95.0	89.3	84.0	89.4	87.0
E	NC	250	92.3	90.8	88.9	90.0	92.4	95.3	89.9	85.6	89.6	87.7
Pooled standard deviation			1.30	1.16	1.93	1.56	1.35	0.98	1.55	1.94	1.66	1.92
*p*-value			<0.001	<0.001	<0.001	<0.001	<0.001	<0.001	<0.001	<0.001	<0.001	<0.001
			-----------------------------------------------Probability--------------------------------------------									
PC vs. NC			<0.001	<0.001	0.004	<0.001	0.005	0.014	<0.001	<0.001	<0.001	<0.001
PC vs. Enzyme-supplemented diets			0.383	0.230	0.058	0.119	0.141	0.118	0.102	0.143	0.830	0.181
Linear effect of enzyme supplementation			0.654	0.481	0.147	0.257	0.278	0.233	0.228	0.306	0.771	0.352
Quadratic effect of enzyme supplementation			<0.001	<0.001	0.001	<0.001	0.002	0.006	<0.001	<0.001	<0.001	<0.001

^1^ PC = Positive control, NC = Negative control. Pre-starter diet fed between d 0 and 9 and starter diet fed between d 9 and 21. ^2^ Number of replicates = 10.

**Table 8:** Effect of increasing level of enzyme supplementation on apparent ileal digestibility of non-essential amino acids in 21-day-old broiler chickens fed corn–soybean meal-based diets (DM basis) ^1,2^.

Treatment	Diet	Allzyme^®^ Spectrum, mg/kg	Ala, %	Asp, %	Cys, %	Glu, %	Gly, %	Pro, %	Ser, %	Tyr, %
A	PC	0	88.4	87.4	80.7	91.8	84.7	87.8	87.2	88.5
B	NC	0	85.2	84.3	74.6	89.7	80.2	84.5	83.1	84.6
C	NC	150	89.0	88.4	81.3	92.5	85.1	88.6	88.1_z_	88.9
D_z_	NC	200	88.7	88.1	80.5	92.3	84.7	88.2	87.4	88.7
E_z_	NC	250	89.3	88.7	81.8	92.7	85.4	88.7	88.0	89.4
Pooled standard deviation			1.62	1.64	2.58	1.23	1.96	1.59	1.50	1.55
*p*-value			<0.001	<0.001	<0.001	<0.001	<0.001	<0.001	<0.001	<0.001
			-----------------------------------------------Probability--------------------------------------------							
PC vs. NC			<0.001	<0.001	<0.001	<0.001	<0.001	<0.001	<0.001	<0.001
PC vs. Enzyme-supplemented diets			0.352	0.120	0.619	0.126	0.645	0.254	0.192	0.227
Linear effect of enzyme supplementation			0.577	0.256	0.965	0.251	0.909	0.439	0.595	0.436
Quadratic effect of enzyme supplementation			<0.001	<0.001	<0.001	<0.001	<0.001	<0.001	<0.001	<0.001

^1^ PC = Positive control; NC = Negative control. Pre-starter diet fed between d 0 and 9 and starter diet fed between d 9 and 21. ^2^ n = 10 replicate cages with 6 birds/replicate, except for subscript z where n = 9.

**Table 9:** Effect of increasing level of enzyme supplementation on tibia quality in 21-day-old broiler chickens fed corn–soybean meal-based diets ^1,2^.

Treatment	Diet	Allzyme^®^ Spectrum, mg/kg	Bone Breaking Strength, kgF	Bone Ash, %
A	PC	0	18.4	51.5
B	NC	0	12.8	48.5
C	NC	150	17.6	51.8
D	NC	200	17.5	51.9_z_
E	NC	250	17.7	51.6
Standard deviation			1.23	1.20
*p*-value			<0.001	<0.001
			--------------------------------Probability----------------------------------	
PC vs. NC			<0.001	<0.001
PC vs. Enzyme-supplemented diets			0.090	0.610
Linear effect of enzyme supplementation			0.091	0.488
Quadratic effect of enzyme supplementation			<0.001	<0.001

^1^ PC = Positive control; NC = Negative control. Pre-starter diet fed between d 0 and 9 and starter diet fed between d 9 and 21. ^2^ n = 10 replicate cages with 6 birds/replicate, except for subscript z where n = 9.

## Data Availability

The data presented in this study are available on request from the corresponding author.
